# Loss of Function in Zeaxanthin Epoxidase of *Dunaliella tertiolecta* Caused by a Single Amino Acid Mutation within the Substrate-Binding Site

**DOI:** 10.3390/md16110418

**Published:** 2018-11-01

**Authors:** Minjae Kim, Jisu Kang, Yongsoo Kang, Beom Sik Kang, EonSeon Jin

**Affiliations:** 1 Department of Life Science, College of Natural Sciences, Hanyang University, Seoul 04763, Korea; sciencekor89@gmail.com; 2 Eugene Biotech Co. Ltd., Daejeon 34051, Korea; eugene2015222@gmail.com (J.K.); romadman@gmail.com (Y.K.); 3 School of Life Science and Biotechnology, Kyungpook National University, Daegu 41566, Korea; bskang2@knu.ac.kr; 4 Research Institute of Natural Sciences, Hanyang University, Seoul 04763, Korea

**Keywords:** zeaxanthin epoxidase, protein structure modeling, marine microalga, *Dunaliella tertiolecta*

## Abstract

The *zea1* mutant of marine microalga *Dunaliella tertiolecta* accumulates zeaxanthin under normal growth conditions, and its phenotype has been speculated to be related to zeaxanthin epoxidase (ZEP). In this study, we isolated the ZEP gene from both wild-type *D. tertiolecta* and the mutant. We found that the *zea1* mutant has a point mutation of the 1337th nucleotide of the ZEP sequence (a change from guanine to adenine), resulting in a change of glycine to aspartate in a highly conserved region in the catalytic domain. Similar expression levels of ZEP mRNA and protein in both wild-type and *zea1* were confirmed by using qRT-PCR and western blot analysis, respectively. Additionally, the enzyme activity analysis of ZEPs in the presence of cofactors showed that the inactivation of ZEP in *zea1* was not caused by deficiency in the levels of cofactors. From the predicted three-dimensional ZEP structure of *zea1*, we observed a conformational change on the substrate-binding site in the ZEP. A comparative analysis of the ZEP structures suggested that the conformational change induced by a single amino acid mutation might impact the interaction between the substrate and substrate-binding site, resulting in loss of zeaxanthin epoxidase function.

## 1. Introduction

Natural products, particularly pharmaceutical and nutraceutical products, have received increasing attention. Many novel marine compounds with pharmaceutical potential have been identified and purified [1], but very few of them are commercially available. Microalgae, single cellular organisms containing diverse natural compounds, are considered to be a sustainable bio-resource for many industrial fields, especially as they have often been investigated for high-valued chemical compounds and health food production [2,3]. For instance, a proper intake of carotenoids (carotene and xanthophylls) is proposed to be beneficial for the proper maintenance of some aspects of human health [4,5]. It has been suggested that carotenoids can prevent or delay cancer and degenerative diseases, including cardiovascular diseases, immune system decline, arteriosclerosis, macular degeneration and cataracts, by contributing to antioxidant defenses against metabolic oxidative byproducts [6,7,8,9].

**Figure 1:**
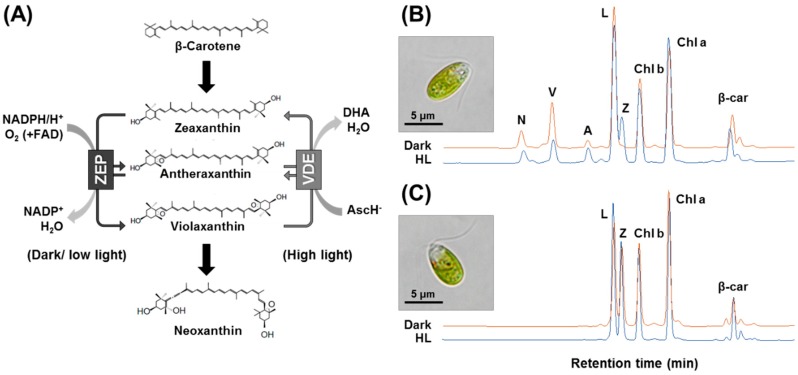
The schematic figure of the xanthophyll cycle (**A**) and the pigment profile of *D. tertiolecta* wild-type (**B**) and *zea1* cells (**C**). The pigment profile was measured after 12 h incubation under dark or high light (HL) conditions. The images of dark-acclimated cells are shown. AscH-, ascorbate monoanion; DHA, dehydroascorbic acid; N, neoxanthin; V, violaxanthin; A, antheraxanthin; L, lutein; Z, zeaxanthin; Chl a, chlorophyll a; Chl b, chlorophyll b; β-car, β-carotene.

Among xanthophylls, β,β-epoxycarotenoids such as zeaxanthin, antheraxanthin, and violaxanthin are important in photosynthetic organisms because they are essential for photosynthesis. These components of the xanthophyll cycle in the photosynthetic apparatus act in photosystem assembly, light harvesting, and photo-protection [10,11]. The xanthophyll cycle requires the activities of zeaxanthin epoxidase (ZEP) and violaxanthin de-epoxidase (VDE) (Figure 1A), which are located on the stromal and luminal side of the thylakoid membrane, respectively [12,13]. Under low light or dark conditions, zeaxanthin is subjected to sequential epoxidation by ZEP to yield antheraxanthin and then violaxanthin. Under excessive light, violaxanthin is converted to antheraxanthin and then zeaxanthin by VDE. The rapid formation of zeaxanthin by the xanthophyll cycle is critical because it prevents photo-inhibition by the reduction of singlet oxygen and dissipation of excess energy as heat through non-photochemical quenching [14,15].

An increased level of zeaxanthin has also been obtained by modifying the carotenoid biosynthetic pathway; the transgenic land plants overexpressing the β-carotene hydroxylase gene show both an increased xanthophyll pigment pool and higher levels of zeaxanthin in high light, which enhanced the resistance to high-light stress [16]. However, some mutants, such as *Arabidopsis thaliana npq2* [17] and *Chlamydomonas reinhardtii npq2* [18], accumulate zeaxanthin under all growth conditions due to a defect in ZEP. These mutants have been used to identify the photo-protective mechanism of the xanthophyll cycle [17,18].

The phenotype of the *zea1* mutant of the marine microalga *Dunaliella tertiolecta*, which was generated by random mutagenesis using ethyl methanesulfonate [19], is similar to that of the *npq2* mutant of *A. thaliana* [20]. This *Dunaliella* mutant has roughly 20-fold higher zeaxanthin content (6 mg per gram dry weight) than the wild type, and does not compete with common land crops with high salinity resistance; thus, it has been proposed as an alternative source for natural zeaxanthin production [20,21]. Nevertheless, the underlying mutation has not been identified yet.

In this study, we have isolated the ZEP gene from marine *D. tertiolecta* (*DtZEP*) and compared its amino acid sequence with ZEPs from various organisms. We verified that *DtZEP* is a single-copy gene through Southern blot analysis. Then, for ZEP from wild-type and the *zea1* mutant, we compared mRNA expression levels, protein content, and enzymatic activity as a function of the cofactor supply. Also, we modeled the ZEP protein structure of wild-type and *zea1* and compared the substrate-binding site through in silico structural analysis. From these results, we suggest that a single mutation in the substrate-binding site of *zea1* causes a conformational change in the substrate-binding site, resulting in the inactivation of ZEP.

## 2. Results and Discussion

### 2.1. Comparison of Pigment Profile between Wild-Type and zea1

We have previously compared the physiological characteristics of *D. tertiolecta* wild-type and *zea1* [20,22,23], but the genotype of *zea1* which results in zeaxanthin accumulation has not been identified. As shown in Figure 1, there is a considerable difference in the pigment profile between the wild type and *zea1*. In the wild type, the peak of zeaxanthin was detected after incubation under high light but not dark conditions, probably as a result of the xanthophyll cycle being affected by high irradiation [24,25]. However, the pigment profile of *zea1* was similar under dark and high light conditions. In a previous study [20], we suggested that the unusual pigment profile of *zea1* might be related to a defect in zeaxanthin epoxidase (ZEP), similar to the *npq2* mutant of *A. thaliana*. Thus, we isolated the DtZEP gene.

### 2.2. Isolation of DtZEP and Sequence Analysis

We isolated the full-length coding DNA sequence of *DtZEP* by PCR with primers designed from the predicted amino acid sequence. The *DtZEP* coding DNA sequence (CDS; 2397 bp) was registered in GenBank (accession number: MH229985). The predicted molecular weight of the encoded protein was 85.09 kDa, the theoretical isoelectric point was 8.20 and instability index was 41.96 [26].

**Figure 2:**
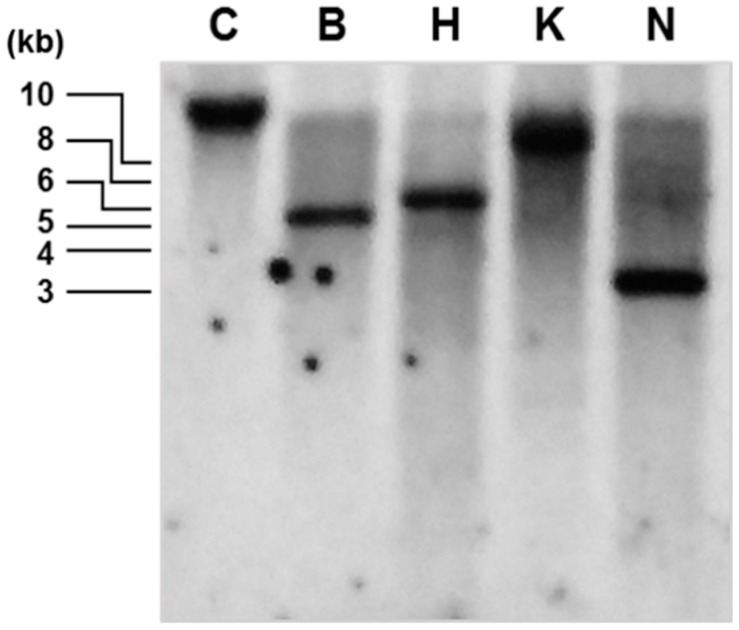
Southern blot analysis. Genomic DNA was digested with *BamHI* (B), *HindIII* (H), *KpnI* (K), or *NcoI* (N). The digested samples and untreated control (C) were separated on a 0.8% agarose gel. A probe of 922 bp amplified from the *DtZEP* gene by PCR was used for hybridization.

To determine the copy number of the ZEP gene in the genome of *D. tertiolecta*, genomic DNA was digested with *BamHI*, *HindIII*, *KpnI*, and *NcoI*, and then a Southern blot analysis was conducted (Figure 2). A single band in each digest indicates the presence of a single copy of the ZEP gene in *D. tertiolecta*, as in other green microalgae [27,28,29] and land plants [17,30,31,32]. However, the diatoms *Phaeodactylum tricornutum* and *Thalassiosira pseudonana* contain three and two copies of the *ZEP* genes, respectively [33,34]. This could be due to the presence of both violaxanthin and diadinoxanthin cycles in these diatoms, whereas the diadinoxanthin cycle is absent in green algae and land plants.

**Figure 3:**
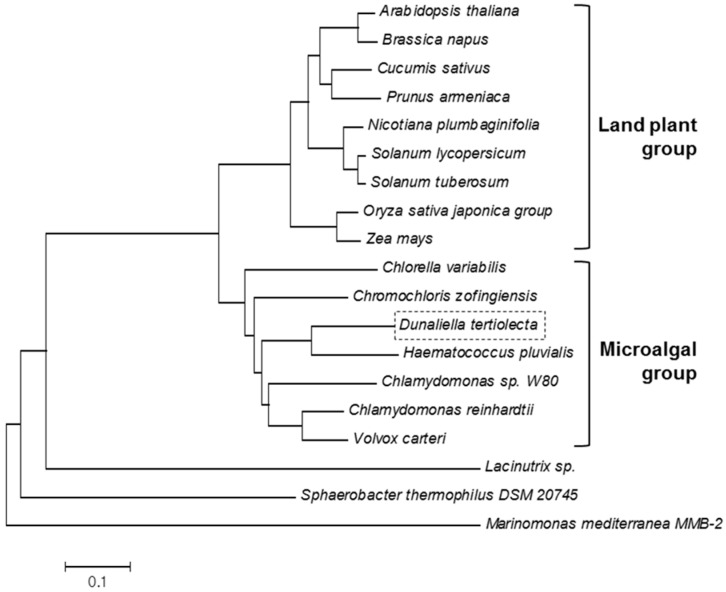
Evolutionary relationships of *D. tertiolecta* zeaxanthin epoxidase (DtZEP) with other ZEPs. The optimal tree (with the sum of branch length = 3.96) is shown. The tree was drawn to scale, with branch lengths in the same units as those of the evolutionary distances used to infer the phylogenetic tree. The analysis involved 18 amino acid sequences. All positions containing gaps and missing data were eliminated. There were a total of 344 positions in the final dataset.

When the deduced amino acid sequence of DtZEP was compared with those of ZEPs from other microalgae, land plants, and bacteria, DtZEP clustered with other microalgal ZEPs as a separate clade in the phylogenetic tree (Figure 3). Expectedly, DtZEP and other microalgal ZEPs are evolutionarily closer to the ZEP genes of land plants than to those of bacteria. These results suggest the functional characteristics of DtZEP might be similar to those of microalgal and land-plant ZEPs.

The results of a BlastP search demonstrated that the DtZEP shared a sequence identity with ZEPs from other microalgal species: 67% with *C. reinhardtii* and *C. zofingiensis*, 63% with *H. pluvialis*, and 55% with *Chlamydomonas* sp. W80. From the alignment of DtZEP and other microalgal ZEPs, we observed a highly conserved region between amino acid residues 141 and 500, which was predicted to be a catalytic domain (Figure S1). This suggests that DtZEP has the same role as other ZEPs, which catalyze epoxidation by using NADPH and FAD as cofactors [35].

### 2.3. Comparative Genotype Analysis of Wild-Type and zea1

**Figure 4:**
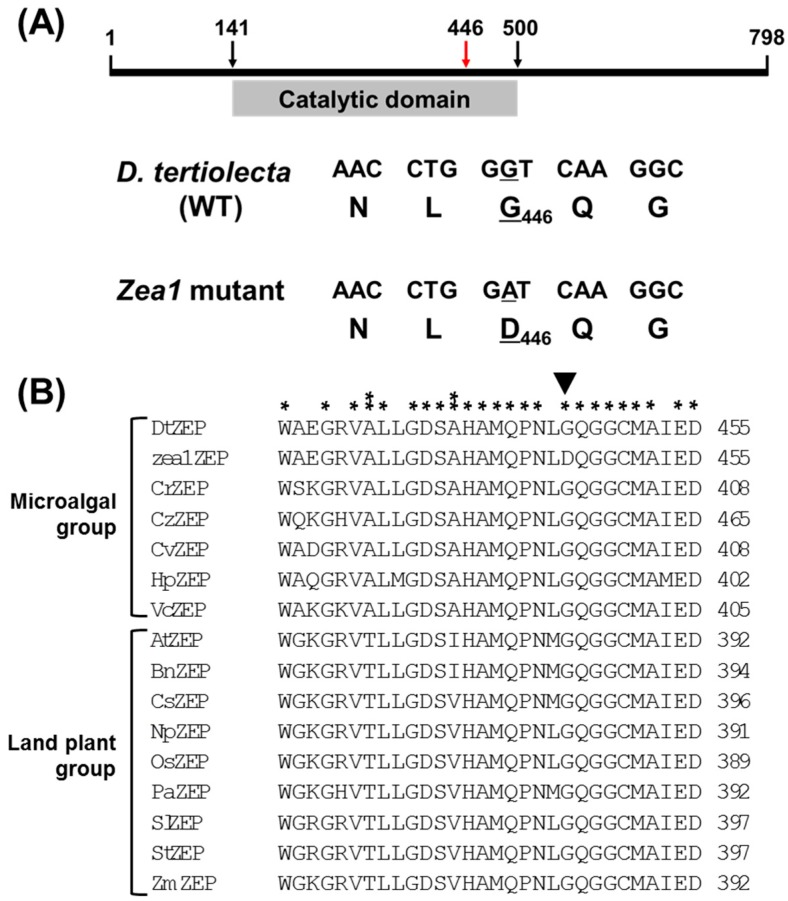
Comparative analysis of the ZEP sequences from wild-type *D. tertiolecta* and *zea1*. (**A**) The structure of DtZEP and position of a point mutation in *zea1*. The mutated amino acid (red arrow, G446D) is located in the catalytic domain (AA 141–500); (**B**) the alignment of the highly conserved amino acid sequences of ZEPs from wild-type *D. tertiolecta*, *zea1*, microalgae, and land plants. Single asterisks indicate amino acids conserved in both microalgal and land-plant ZEPs; double asterisks indicate amino acids conserved only among microalgal ZEPs. The black triangle shows the position of the point mutation. Sequences from the following species were compared: DtZEP, *D. tertiolecta* wild type; *zea1*ZEP, *D. tertiolecta zea1*; CrZEP, *Chlamydomonas reinhardtii*; CzZEP, *Chromochloris zofingiensis*; CvZEP, *Chlorella variabilis*; HpZEP, *Haematococcus pluvialis*; VcZEP, *Volvox carteri*; AtZEP, *Arabidopsis thaliana*; BnZEP, *Brassica napus*; CsZEP, *Cucumis sativus*; NpZEP, *Nicotiana plumbaginifolia*; OsZEP, *Oryza sativa*, *japonica* group; PaZEP, *Prunus armeniaca*; SlZEP, *Solanum lycopersicum*; StZEP, *Solanum tuberosum*; and ZmZEP, *Zea mays*.

We compared the deduced amino acid sequences of ZEPs from wild-type *D. tertiolecta* and *zea1*. An alignment of CDS showed that *zea1ZEP* has a single nucleotide change at 1337 bp (guanine to adenine), resulting in a single amino acid change from glycine to aspartate (Figure 4A).

We compared the highly conserved region (AA 426–455) of the catalytic domain in the ZEPs of wild-type *D. tertiolecta* and *zea1* with other microalgal and land-plant ZEPs. Most amino acids in the domain were conserved among microalgal and land-plant ZEPs (* in Figure 4B), but there were some differences between microalgal and land-plant ZEPs (** in Figure 4B). Because this region is highly conserved, it seems to be very important for the function of this enzyme in both microalgae and land plants (* in Figure 4B). Therefore, the substitution of the conserved glycine in *zea1* might lead to the structural modification of the ZEP protein, perhaps because the glycine to aspartate change could alter local conformation and the electrical field strength in the conserved regions of the catalytic domain.

### 2.4. A Single Amino Acid Change Does Not Affect the Levels of ZEP mRNA and Protein in zea1

**Figure 5:**
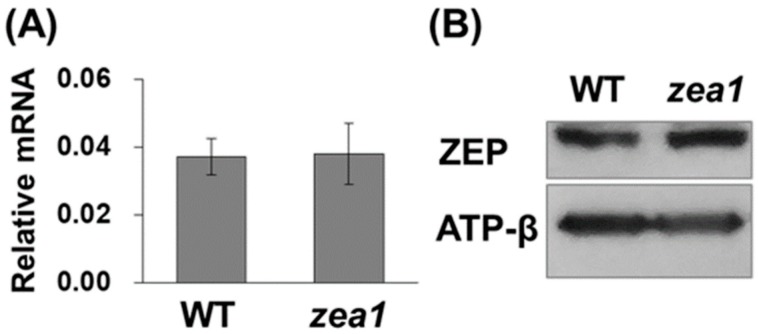
Expression levels of ZEP in wild-type *D. tertiolecta* and *zea1*. (**A**) Transcript levels calculated relative to those of Dt60S used as a reference; (**B**) protein levels of ZEPs. The expected protein size of ZEP was detected (about 85 kDa), and the β-subunit of ATP synthase (56 kDa) was used as the loading control. All experiments were conducted in triplicate.

Although there is no report about the mRNA and protein expression levels of *A. thaliana npq2* and *C. reinhardtii npq2* [17,18], it is known that recent ZEP-targeted knock-out *C. reinhardtii* mutants do not synthesize a ZEP protein due to a large insertional mutation [36]. In contrast to these ZEP-knock-out *C. reinhardtii* mutants, *zea1* has a single amino acid change that does not result in a large insertional mutation, and so we expected that mRNA and protein expression levels may not be different in *zea1*, compared with wild-type. To verify this hypothesis, we investigated ZEP mRNA expression in the wild type and *zea1* using quantitative real-time PCR. As expected, the ZEP mRNA level was almost the same in *zea1* and the wild type (Figure 5A). Likewise, similar levels of the ZEP protein were detected by Western blot analysis (Figure 5B). Similar levels of mRNA and protein expression with wild-type indicated that the zeaxanthin accumulation of *zea1* did not result from abnormal ZEP gene expression. Therefore, we hypothesized that the zea1 phenotype results from the dysfunction of ZEP.

### 2.5. Additional Cofactor Supply Does Not Affect the Enzymatic Activity of ZEP in zea1

**Figure 6:**
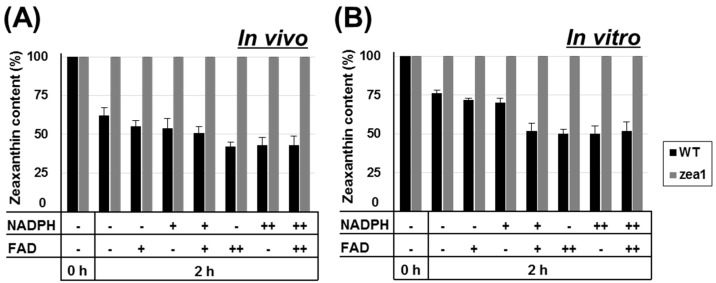
Zeaxanthin epoxidase activity with and without the supplementation of additional cofactors. The zeaxanthin content of the wild type and *zea1* was determined relative to control (0 h) in the presence or absence of cofactors. (**A**) In vivo assay; (**B**) in vitro assay. NADPH and FAD were added at 0 mM (-), 10 mM (+), or 100 mM (++). All experiments were conducted in triplicate.

NADPH and FAD are essential cofactors for the sequential epoxidation of zeaxanthin by ZEP [13,35]; zeaxanthin + 2NAD(P)H + 2H^+^ + 2O_2_→violaxanthin + 2NAD(P)^+^ + 2H_2_O (electron transfer aided by FAD) (Figure 1A). These cofactors are also required for electron transfer during photosynthesis [37], and insufficient levels of these cofactors of ZEP could hamper photosynthesis [38]. Our previous studies demonstrated that the inactivation of ZEP had no measurable impact on the growth and photosynthesis of *zea1* [20,23], and that the photosynthetic efficiency of *zea1* is similar to that of the wild type [39]. These results indicate that levels of NADPH and FAD in cells are not depleted in *zea1* and are sufficient for normal photosynthesis and cell growth. Nevertheless, the effect of cofactors on ZEP activity has not been previously investigated in wild type and *zea1*. To assay ZEP activity, we measured zeaxanthin content, which should be decreased due to being converted into violaxanthin. We added NADPH and/or FAD into culture medium (in vivo assay) and cell lysate (in vitro assay). In case of the in vivo assay, the zeaxanthin content of both the control and experimental group in wild-type decreased after 2 h of incubation in the dark. An additional supply of cofactors increased the activity of ZEP by 11% (with 10 mM of cofactors) and 19% (100 mM of cofactors) in wild-type samples. However, zeaxanthin content of *zea1* samples remained constant regardless of the cofactors added (Figure 6A). Similar results were obtained in vitro using cell lysate (Figure 6B). An excess supply of cofactors did not lead to the recovery of ZEP activity in *zea1* samples, suggesting that the loss of function of ZEP in *zea1* is due to a mutation in the ZEP protein. To investigate the protein structures, three-dimensional models of ZEP from both wild type and *zea1* were constructed and compared.

### 2.6. Comparison of the Three-Dimensional ZEP Structures of Wild-Type and zea1

Hereafter, we refer to ZEP of *zea1* as ZEP_D446_ and to ZEP of the wild type as ZEP_G446_. Using ProtParam [40], we predicted the molecular weights of ZEP_G446_ (85.09 kDa) and ZEP_D446_ (85.15 kDa) and their isoelectric points (pI values; 8.20 and 8.02, respectively). The single amino acid mutation in ZEP_D446_ increased the total number of negatively charged residues, and so we expected a conformational change in the mutant protein structure of ZEP_D446_. Initially, we generated homology models using SWISS-model server based on the oxidoreductases recommended as template structures to investigate the structural differences in the enzymatic active site between ZEP_G446_ and ZEP_D446_.

**Figure 7:**
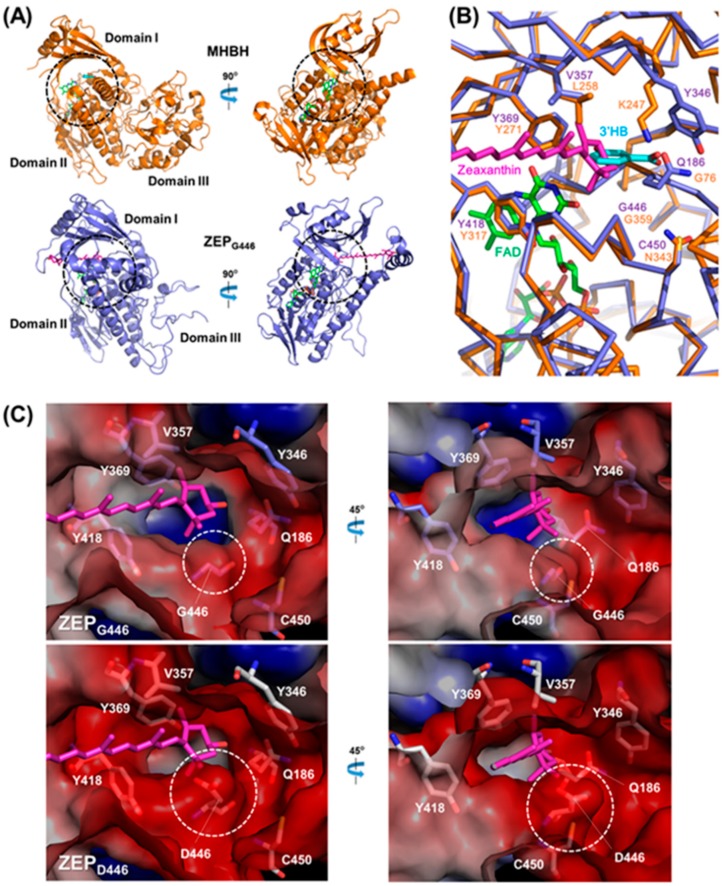
Comparative analysis of predicted three-dimensional structures of ZEP_G446_ (wild type) and ZEP_D446_ (*zea1*). (**A**) Ribbon diagram of the overall structure of ZEP_G446_ (lavender) and the 3′-hydroxybenzoate hydrolase (MHBH) (orange). The ZEP_G446_ model structure consists of the catalytic domain (domain I and domain II) and the C-terminal domain (domain III). Views rotated counterclockwise by 90° are shown on the right. The substrate-binding site is indicated by a black dashed circle; (**B**) close-up view of the substrate-binding site. Amino acid residues involved in substrate binding are shown as stick models; (**C**) close-up view of the tunnel around the substrate-binding site represented by the electrostatic potential surface. Views rotated counterclockwise by 45° are shown on the right. Amino acid residues involved in substrate binding are shown as stick models with different colors (lavender: ZEP_G446_, white: ZEP_D446_). Each color for the electrostatic potential of protein surface indicates negative charge (red), neutral charge (white), and positive charge (blue). The white dashed circle indicates a protruded region on ZEP_D446_. For this analysis, the crystal structure of 3′-hydroxybenzoate hydroxylase from *C. testosteroni* (PDB ID: 2DKH) was used for the template. FAD, zeaxanthin (substrate of ZEP), and 3′-hydroxylbenzoate (substrate of MHBH) are represented as stick models and colored bright green, hot pink, and cyan, respectively.

The final ZEP_G446_ protein model was generated by conjugating two protein models: the main part of the active site (to the 491st amino acid) was built with the model based on 3′-hydroxylbenzoate hydroxylase (MHBH) (PDB ID: 2DKH) and the C-terminal part of the active site (from the 492nd amino acid) was built with the model based on Baeyer–Villiger monooxygenase (PDB ID: 3FMW). The protein fold of the substrate-binding site in the catalytic domain was predicted to form a tunnel structure (Figure S2), commonly found in many oxidoreductases, such as hydroxylase and oxygenase [41,42,43]. The structure of the catalytic domain in the predicted ZEP_G446_ model is similar to that of its template, MHBH of *Comamonas testosteroni* (domain I and II), although the C-terminal domain of the model is different from that of the MHBH (Figure 7A). When the substrate-binding site of ZEP_G446_ was compared to that of the MHBH (Figure 7B), the conserved residues such as Y418 and Y369 (Y317 and Y271 of the MHBH, respectively) were thought to be essential for interacting with FAD [42]. A conserved glycine, G446 (G359 of MHBH) provides the space for a substrate. The residues in the active site such as V357, Y346, Q186, and C450, which have different side chain to the MHBM (L258, K247, G76, and N343 of MHBH, respectively), were expected to interact with its substrate. Interestingly, these amino acids were highly conserved on the microalgal ZEPs (Figure S1). From this result, we expected that these highly conserved amino acids are important to bind zeaxanthin in the active site of ZEPs. Next, zeaxanthin was superimposed to the active site: a ring moiety of zeaxanthin was located at the position of 3′-hydroxylbenzoate (Figure 7B) and the aliphatic chain was located at the tunnel region (Figure S2).

To investigate the effect of single amino acid mutation on ZEP_D446_ to the substrate-binding site, we visualized the electrostatic potential of the protein surface and compared the tunnel region of ZEP_G446_ and ZEP_D446_ (Figure 7C). These results reveled a conformational change near the mutated amino acid. The active site of ZEP_G446_ has a flat region for substrate binding, but the active site of ZEP_D446_ has a protruded region formed by a carboxylic acid group (-COOH) of 446th aspartate. To quantify a difference in this region, we measured the solvent-accessible surface area of the protruded region (AA 443–446), the distance between the residues surrounding the substrate-binding site (Figure S3), and the volume of substrate pocket cavity (Figure S4). The surface areas around the protruded region were 900 Å^2^ and 944 Å^2^ on ZEP_G446_ and ZEP_D446_, respectively. The shortest distances between the 357th amino acid and 446th amino acid were 9.1 Å and 6.9 Å on ZEP_G446_ and ZEP_D446_, respectively. Also, the volumes of substrate pocket cavity were 2556 Å^3^ and 2,375 Å^3^ on ZEP_G446_ and ZEP_D446_, respectively. The small cavity volume of ZEP_D446_ seems to be caused by the bulged area resulting from the bulky side chain of aspartate. Therefore, considering the van der Waals volume of the substrate, the protruded region of ZEP_D446_ could affect substrate binding.

In addition to the structural change, the negatively charged side chain of the 446th aspartate strongly changed the electrostatic potential of the active site to a negative charge. For an enzyme reaction to occur, the charge distribution of the substrate and the active site must be complementary, which means that all positive and negative charges must be canceled [44,45]. Because the substrate of ZEP is a polar carotenoid pigment that has no charge, the strongly negatively charged region of ZEP_D446_ can affect the interaction between substrate and substrate-binding site.

Tighter interactions between the substrate and the substrate-binding site are essential for most enzymatic reactions [46,47]. However, the protruding regions of strongly negative ZEP_D446_ may have difficulty interacting with the substrate. As a result of the in silico analysis, we conclude that conformational changes in the active site of ZEP_D446_ can interfere with substrate binding, resulting in the inactivation of the enzyme. However, it is thought that further structural analysis of ZEPs using the crystal structure and/or site-specific mutations of the recombinant protein is necessary to reveal precise interactions between the substrate and substrate-binding sites on the ZEP.

## 3. Materials and Methods

### 3.1. Strains and Culture Conditions

The wild-type *Dunaliella* strain used in our studies was initially thought to be *D. salina* but was recently reclassified as *D. tertiolecta* on the basis of its genomic and physiological characteristics [23]. The *zea1* mutant strain, which cannot synthesize any β,β-epoxycarotenoids derived from zeaxanthin, is described in the Introduction. It constitutively accumulates zeaxanthin, but lacks antheraxanthin, violaxanthin, and neoxanthin under all growth conditions. Cells were grown photoautotrophically in 250 mL flasks under continuous white fluorescence lighting (50 ± 5 μmol photons m^–2^ s^–1^; low-light conditions) at 25 ± 1 °C. Cultures were shaken continuously on an orbital shaker (90 rpm). The nutrient composition of growth medium (D medium) was described in our previous paper [23].

### 3.2. Cloning of Zeaxanthin Epoxidase Gene

The sequence of the zeaxanthin epoxidase (ZEP) gene of *D. tertiolecta* was predicted using the *D. salina* CCAP 19/18 database (*D. salina* Genome Sequencing Project; http://phytozome.jgi.doe.gov/; [48]) and the transcriptome database of *D. tertiolecta* (Marine Microbial Eukaryote Transcriptome Sequencing Project, [49]). Primers for the amplification of the *DtZEP* open reading frame were designed on the basis of the predicted sequence (forward, 5′-ATGCTGCGCAGTGCCCTGAGCGGCT-3′; reverse, 5′-TTATTGGGCCTGCACCATTGCCCCTGAG-3′). The coding DNA sequence was amplified from the complementary DNA (cDNA) synthesized using Pfu Plus 5× PCR Master Mix (ELPiS Biotech, Daejeon, Korea).

### 3.3. Sequence and Phylogenetic Analysis

The molecular weight, isoelectric point, and instability index of DtZEP were estimated in the ProtParam program [26]. Sequence analysis and alignment were performed using the Clustal W algorithm of MEGA6 [50]. The sequences were retrieved from the NCBI GenBank. Based on the alignment, a phylogenetic tree was constructed using MEGA6 [50] and the neighbor-joining method [51]. The evolutionary distances corresponded to the number of amino acid substitutions per site, and were computed using the Poisson correction method [52]. The names of the species (and their GenBank accession numbers) included in the analysis were as follows: *Arabidopsis thaliana* (AAG17703.1), *Brassica napus* (ADC29517.1), *Chlamydomonas* sp. W80 (AAO48941.1), *Chlamydomonas reinhardtii* (XP_001701701.1), *Chlorella variabilis* (EFN52633.1), *Chromochloris zofingiensis* (CCI79384.1), *Cucumis sativus* (ADP69105.1), *Haematococcus pluvialis* (AKT95177.1), *Lacinutrix* sp. (AEH02389.1), *Marinomonas mediterranea* MMB-2 (ADZ93016.1), *Nicotiana plumbaginifolia* (CAA65048.1), *Oryza sativa*, *japonica* group (BAB39765.1), *Prunus armeniaca* (AAD42899.1), *Solanum lycopersicum* (ABQ52698.1), *Solanum tuberosum* (ADF28629.1), *Sphaerobacter thermophilus* DSM 20745 (ACZ40773.1), *Volvox carteri* (XP_002953670.1), and *Zea mays* (ACG42893.1).

### 3.4. Isolation of Genomic DNA and Southern Blot Analysis

Genomic DNA was extracted from *D. tertiolecta* using a protocol modified from Tanksley et al. [53]. Genomic DNA (20 µg) was digested with different restriction enzymes (*BamHI*, *HindIII*, *KpnI*, and *NcoI*, TaKaRa, Kusatsu, Japan). The fragments were separated on a 0.8% agarose gel, transferred onto a positively charged nylon membrane (Hybond-N^+^, GE Healthcare, Chicago, IL, USA), and cross-linked using shortwave ultraviolet light (254 nm). The probe was designed to cover 922 bp of the exon and intron sequence of *DtZEP*, and was amplified from genomic DNA by PCR with the forward primer 5′-GCGTCTGACAGGTGTGTCTG-3′ and reverse primer 5′-GCACATCGGACACAACTCTG-3′. The amplified probe was labeled with alkaline phosphatase using the Gene Images AlkPhos Direct Labeling and Detection System kit (GE Healthcare, Chicago, IL, USA). Labeling, hybridization, washing, and signal detection were conducted according to the manufacturer’s protocol.

### 3.5. Quantitative Real-Time PCR

Total RNA was extracted with an RNeasy Plant Mini Kit (Qiagen, Hilden, Germany) from cells in the mid-exponential phase. cDNA was synthesized from total RNA by using 2× reverse transcription master premix (ELPiS Biotech, Daejeon, Korea) and amplified using SYBR premix (TaKaRa, Kusatsu, Japan) in a Thermal Cycler Dice Real Time System TP 8200 (TaKaRa, Kusatsu, Japan). The gene for the 60S ribosomal protein large subunit (Dt60S) was used as a reference and was amplified with the forward primer 5′-CCAAGCTCACCTACGAGCAG-3′ and reverse primer 5′-GTCCACCAACCCGAATCCAT-3. The *ZEP* transcript was amplified with the forward primer 5′-ACAAGCATGGTACGGGGATG-3′ and reverse primer 5′-CATGGCCTGCATGACCACTA-3′. The results were analyzed by the ΔΔCt method.

### 3.6. Western Blot Analysis

Equal amounts of crude protein extracts (on the basis on equal cell numbers; 100 × 10^4^ cells per lane) were obtained by homogenization in 20 mM HEPES-KOH (pH 7.5), 5 mM MgCl_2_, 5 mM β-mercaptoethanol, and 1 mM phenylmethylsulfonyl fluoride (PMSF, serine protease inhibitor) and were separated by electrophoresis in 8% SDS-polyacrylamide gels [54]. The gels were stained with 0.1% (*w*/*v*) Coomassie Brilliant Blue R or blotted onto polyvinylidene fluoride membranes in a semi-dry transfer system. The membranes were probed with custom-made polyclonal antibody against ZEPs of *D. tertiolecta*. This antibody was raised in a rabbit using the synthetic peptides EGCITGDRINGLCDG (100% identity to DtZEP) and DLIKATPEEDVLRRDIFDR (84% identity to DtZEP) as antigens. The β-subunit of ATP synthase (ATP-β) (Agrisera, Vännäs, Sweden) was used as the control protein. The horseradish peroxidase conjugated goat anti-rabbit IgG (H+L) antibody (Invitrogen, Carlsbad, CA, USA) was used as secondary antibody. Signals were visualized using the PicoEPD Western Reagent (ELPiS Biotech, Daejeon, Korea).

### 3.7. Treatment with ZEP Cofactors

We modified the methods described previously [12,55]. Cells were cultured under high-light conditions (300 ± 10 μmol photons m^–2^ s^–1^) for 12 h to accumulate zeaxanthin. Solutions of two ZEP cofactors, NADPH and FAD, were prepared at 10 mM and 100 mM. For in vivo assay, cofactors were added into the culture medium. For in vitro assay, cofactors were added into the crude cell extract, which was prepared as follows. (i) Cells were harvested by centrifugation (14,000× *g*, 2 min) and resuspended in 330 mM sorbitol, 10 mM NaCl, 5 mM MgCl_2_, and 40 mM HEPES (pH 7.5). (ii) The cells were crushed by vortexing with glass beads for 1 min. (iii) After the glass beads sank, the supernatant was used as crude extract (cell lysate). After cofactor addition, the samples were incubated in the dark to allow ZEP reaction. The pigment extraction process was different according to the assay methods. For the sample in the in vivo assay, cells were harvested by centrifugation (14,000× *g*, 2 min) and resuspended with 0.5 mL of 90% (*w*/*w*) acetone. For the sample in the in vitro assay, nine volumes of 100% (*w*/*w*) acetone was added to one volume of cell lysate to make 90% (*w*/*w*) acetone and resuspended. The zeaxanthin content was expressed using relative ratio to control (0 h). Three independent measurements were performed.

### 3.8. Xanthophyll Composition Analysis

Each 0.5 mL sample (2.5 × 10^6^ cells mL^−1^) was harvested by centrifugation (14,000× *g*, 2 min), and the supernatant was discarded. Xanthophyll pigments were extracted from the pellet using 0.5 mL of 90% (*w*/*w*) acetone for 1 min. After centrifugation at 14,000× *g* for 5 min (at 4 °C to prevent pigment degradation), the supernatant was filtered through a 0.2-μm nylon filter and was analyzed by using a Shimadzu Prominence high-performance liquid chromatography (HPLC) system (model LC-20AD) equipped with a Waters Spherisorb S5 ODS2 cartridge column (4.6 × 250 mm). Xanthophyll pigments were identified by retention time and absorption spectra with reference to pigment standards DHI C14 (DHI, Centralen, Denmark). Details of HPLC analysis have been described in Park et al. [56].

### 3.9. Prediction of Protein Structure by Comparative Modeling

To predict the ZEP structures of the wild type and *zea1*, homology (comparative) modeling was performed. For the modeling of wild-type (ZEP_G446_) and *zea1* (ZEP_D446_), the SWISS-Model server [57] was used. Two templates were used to construct *Dunaliella* ZEPs: one is the crystal structure of 3′-hydroxybenzoate hydroxylase from *Comamonas testosteroni* (PDB ID: 2DKH) and the other is the crystal structure of Baeyer–Villiger monooxygenase from *Streptomyces argillaceus* (PDB ID: 3FMW). The final protein model was generated by conjugating two protein models: the main part of the active site (to the 491th amino acid) was built with the model based on 3′-hydroxylbenzoate hydroxylase and the C-terminal part of the active site (from the 492th amino acid) was built with the model based on Baeyer–Villiger monooxygenase. The putative protein model was analyzed and visualized using PyMOL 2.0 (http://www.pymol.org). Also, the measurement for quantification was performed using PyMOL and 3V [58]: the surface areas for the solvent-accessible parts of the protruded region and the distance between the residues surrounding the substrate-binding site (PyMOL), and the volume of substrate pocket cavity (3V).

## 4. Conclusions

To unveil the molecular features of *zea1* that lead to zeaxanthin accumulation but no other β,β-epoxycarotenoids, we isolated the ZEP gene in marine microalga *D. tertiolecta* and confirmed that this species has a single copy of the ZEP gene. As a result of the comparative sequence analysis of the ZEP gene, the ZEP gene of *zea1* has a point mutation at the 1337th nucleotide of the ZEP gene resulting in a change from glycine to aspartate in the catalytic domain (AA 141-500). This single point mutation did not affect the expression level of ZEP mRNA or protein, but instead displayed a complete loss of activity even when excess supplies of cofactors were provided. To determine the effect of single amino acid changes of ZEP on its enzymatic activity, we predicted and compared the three-dimensional structure of ZEP from wild-type and *zea1*. A comparative analysis of these predicted ZEP structures revealed that a change of glycine to aspartate in the active site resulted in a protrusion of the tunnel of substrate-binding site in ZEP_D446_. Despite sufficient substrate and cofactor levels, the bulky side chains of the aspartate of ZEP_D446_ can interfere with substrate binding. Additionally, the negatively charged aspartate of ZEP_D446_ can interfere with the interaction between the substrate and substrate-binding site. Taken together, we conclude that the mutated site in ZEP_D446_ may result in the loss of function on ZEP in *zea1* via interference with substrate binding; however, further investigation of the structural analysis through X-ray crystallography or amino acid substitution analysis for the structure–activity relation is needed.

## Supplementary Materials

The following are available online at http://www.mdpi.com/1660-3397/16/11/418/s1.
