# Supplementary material for: Loss of Function in Zeaxanthin Epoxidase of Dunaliella tertiolecta Caused by a Single Amino Acid Mutation within the Substrate-Binding Site
Source: Mar Drugs. 2018 Nov 1;16(11):418. doi: 10.3390/md16110418 (PMC6266236; doi:10.3390/md16110418)
Supplement: Supplementary file 1 [file marinedrugs-16-00418-s001.pdf]

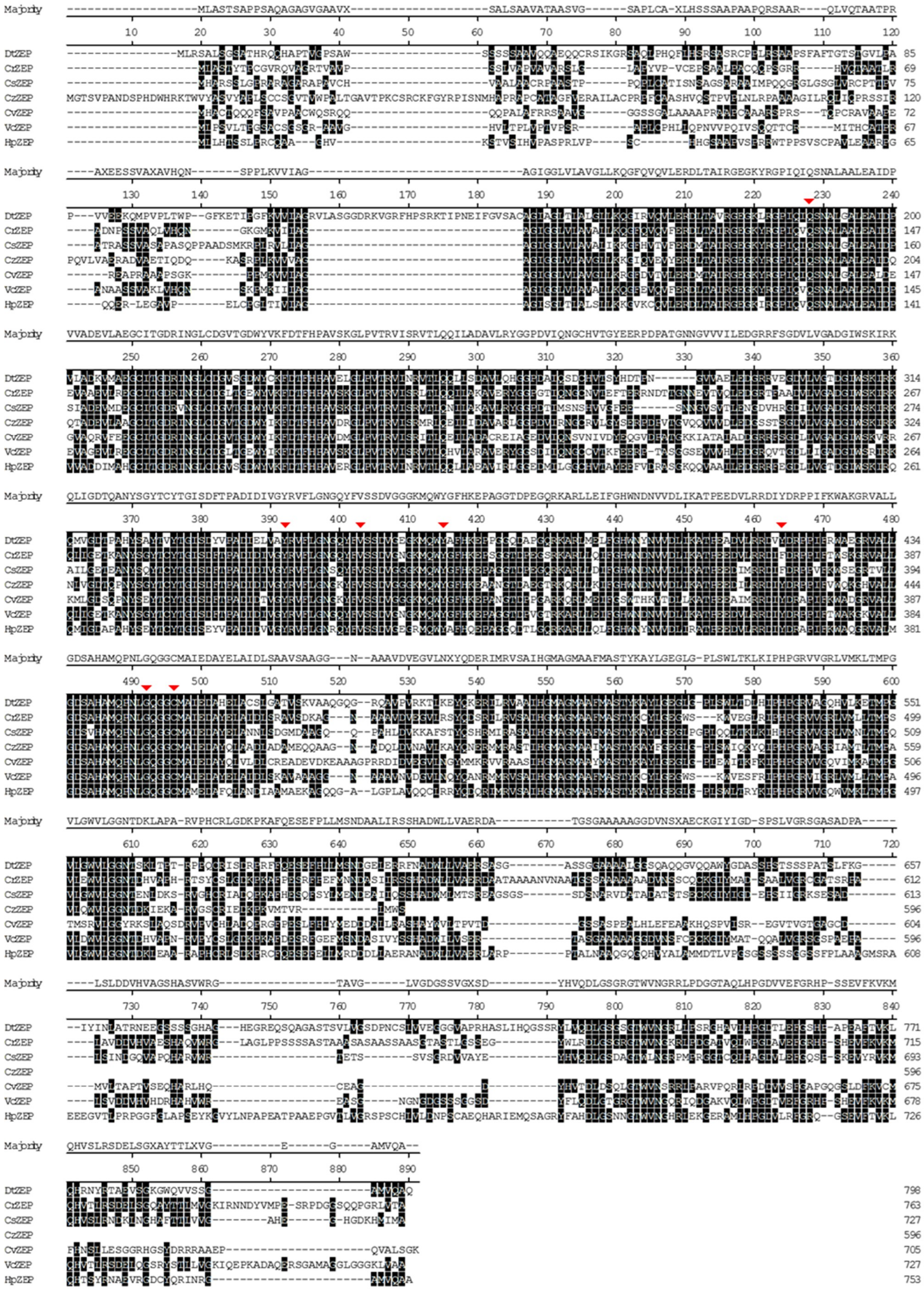
Majority
DtZEP
CrZEP
CsZEP
CzZEP
CvZEP
VcZEP
HpZEP

Supplementary Figure 1. Alignment of amino acid sequences of DtZEP and other microalgal ZEPs. A highly conserved region was predicted as the FAD-binding domain (AA 141–500, DtZEP numbering). The amino acids pointed by red inverted triangle were thought that their side chain involved to substrate-binding site (Q186, Y346, V357, Y369, Y418, G446, and C450, DtZEP numbering). Sequences of ZEPs from the following species were used: DtZEP, *D. tertiolecta* (identified in this study); CrZEP, *Chlamydomonas reinhardtii* (XP_001701701.1); CsZEP, *Chlamydomonas* sp. W80 (AAO48941.1); CzZEP, *Chromochloris zofingiensis* (CCI79384.1); CvZEP, *Chlorella variabilis* (EFN52633.1); VcZEP, *Volvox carteri* (XP_002953670.1); HpZEP, *Haematococcus pluvialis* (AKT95177.1).

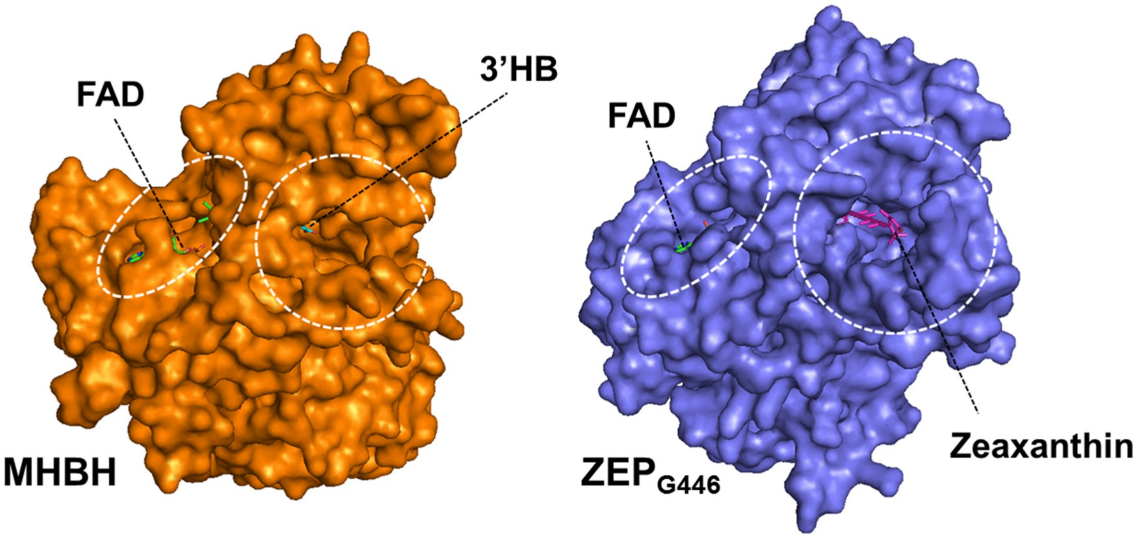

Supplementary Figure 2. Surface diagram of modeled ZEP$_{G446}$ (lavender) and its modeling 3'-hydroxybenzoate hydroxylase (MHBH) (orange). The white dashed circles indicate the tunnel for substrate-binding pocket. The substrates which are 3'-hydrobenzoate (3'-HB) of template and zeaxanthin of ZEP$_{G446}$, are located inside the right pocket. And FAD is located inside the left pocket. The template structure is the crystal structure of 3'-hydroxybenzoate hydroxylase from *Comamonas testosteroni* (PDB ID: 2DKH).

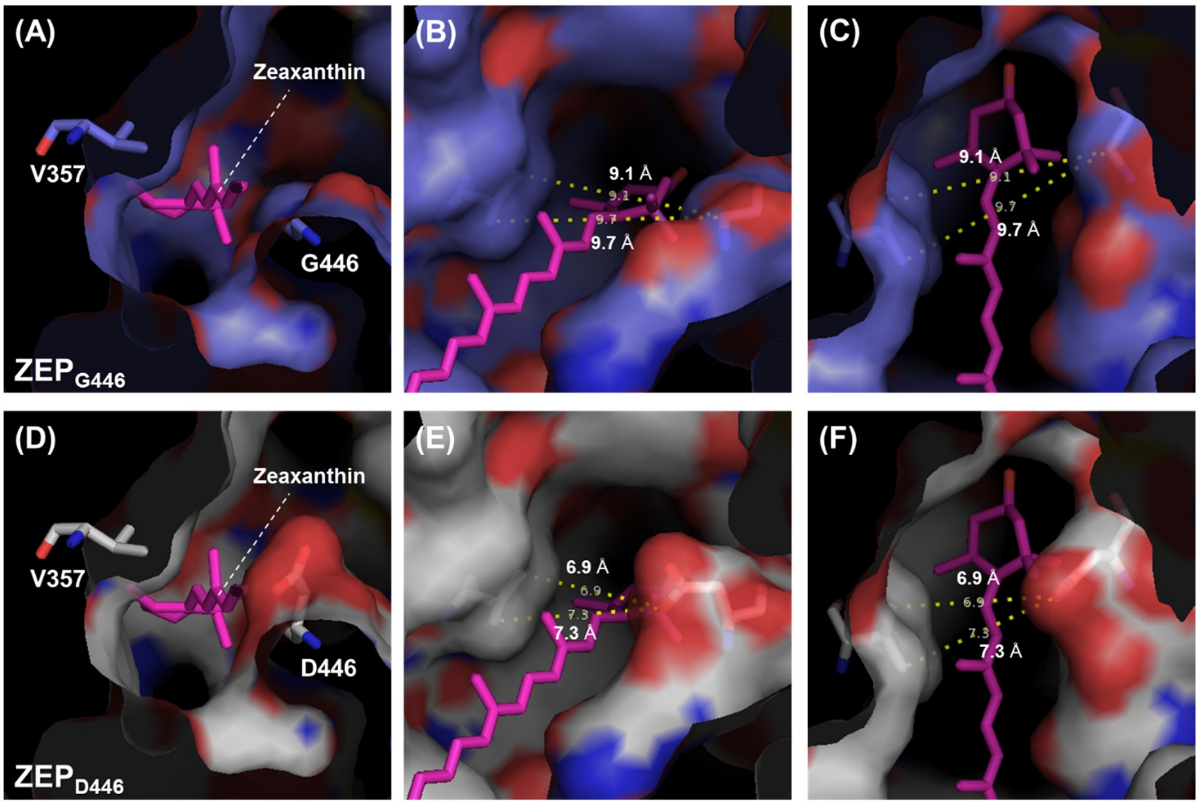

Supplementary Figure 3. Close-up view of zeaxanthin and the tunnel region on substrate-binding site. (A) Cross section view of tunnel region on ZEP_{G446}; (B, C) The distance between G446 and an amino acid at the opposite wall of the tunnel (V357); (D) Cross section view of tunnel region on ZEP_{D446}; (E, F) The distance between D446 and an amino acid at the opposite wall of the tunnel (V357). The distance was measured by PyMOL program.

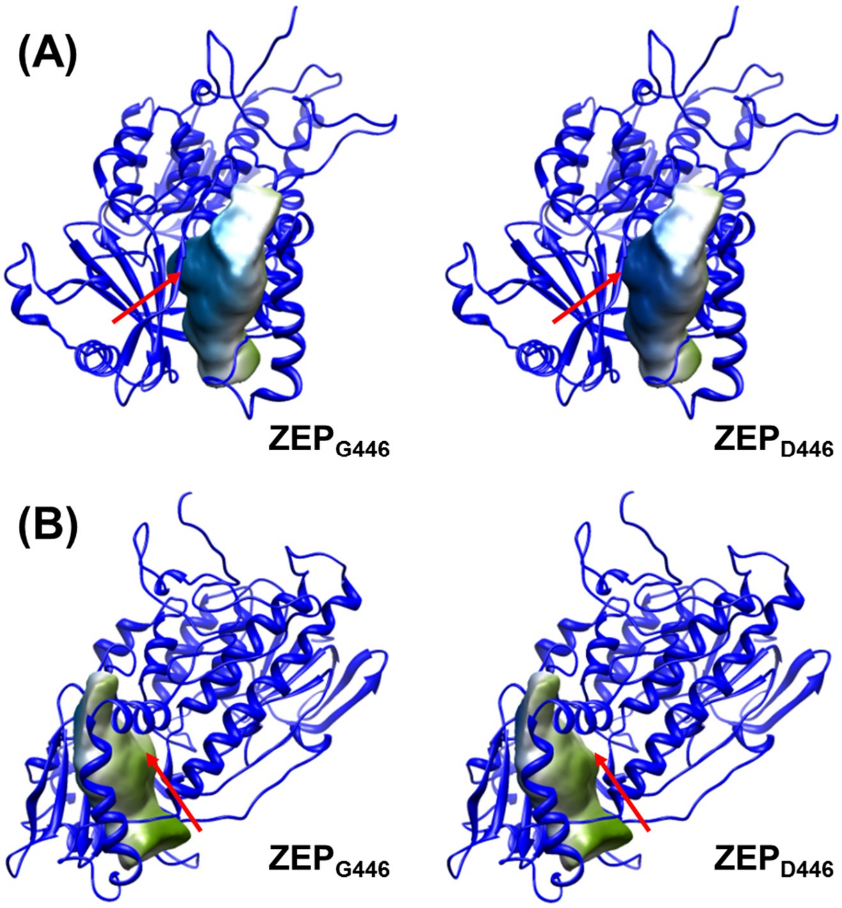

Supplementary Figure 4. The substrate pocket cavity of ZEP_{G446} and ZEP_{D446}. (A) and (B) present different sides of this model structure. Red arrows highlight different regions of the substrate pocket cavity between ZEP_{G446} and ZEP_{D446}. The volume of substrate pocket cavity was analyzed using the 3V program.
